# FABP4 inhibitor BMS309403 protects against hypoxia‐induced H9c2 cardiomyocyte apoptosis through attenuating endoplasmic reticulum stress

**DOI:** 10.1111/jcmm.15666

**Published:** 2020-09-07

**Authors:** Fuqiang Sun, Jiangchuan Du, Hongbin Li, Shuang Hao, Guochang Zhao, Fanfan Lu

**Affiliations:** ^1^ Department of Cardiovascular Surgery The First Affiliated Hospital of Zhengzhou University Zhengzhou China; ^2^ Department of Ultrasound The First Affiliated Hospital of Zhengzhou University Zhengzhou China; ^3^ Department of Critical Care Medicine The First Affiliated Hospital of Zhengzhou University Zhengzhou China

**Keywords:** apoptosis, BMS309403, cardiomyocytes, endoplasmic reticulum stress, FABP4, hypoxia

## Abstract

Acute myocardial infarction is characterized by ischaemia‐induced cardiomyocyte apoptosis, in which the endoplasmic reticulum (ER) stress plays an important role. The fatty acid‐binding protein‐4 (FABP4) has been implicated in regulating ER stress and apoptosis. Yet, whether FABP4 is involved in modulating cardiomyocyte apoptosis remains unclarified. By applying an in vitro model of hypoxia‐induced apoptosis of H9c2 cardiomyocytes, we found that FABP4 expression was elevated upon hypoxia stimulation, which was further demonstrated to be transcriptionally activated by the hypoxia‐inducible factor 1a (HIF‐1α). In addition, the pharmacological inhibition of FABP4 with BMS309403 protected against hypoxia‐induced apoptosis in cardiomyocytes, indicating that FABP4 induction is detrimental for cardiomyocyte survival under hypoxic condition. Moreover, BMS309403 attenuated ER stress in cardiomyocytes exposed to hypoxia, which, however, was reversed by tunicamycin, an ER stress activator. More importantly, the protective effect of BMS309403 on cardiomyocytes vanished in the presence of tunicamycin. Thus, these observations establish that FABP4 inhibitor BMS309403 reduces hypoxia‐induced cardiomyocyte apoptosis through attenuating excessive ER stress, implying that FABP4 inhibition may be of clinical benefit for MI treatment.

## 1 INTRODUCTION

Acute myocardial infarction (MI), one of the leading causes of disability and death in the world,^1^ is featured by the ischaemia‐induced cardiomyocyte loss due to necrosis and apoptosis.^2^ To date, increasing evidence has shown that the cardiomyocyte apoptosis spotted in the border zone of infarct lesions and the remote zone of non‐infarcted myocardium deteriorates the post‐MI remodelling and cardiac dysfunction, together contributing to heart failure development.^3^, ^4^ Therefore, advancing the mechanisms of cardiomyocyte apoptosis during the early stage of MI is extremely important for reversing this pathogenic process and ameliorating cardiac function of patients with MI.

In recent years, a growing body of studies has noted that the dysregulated endoplasmic reticulum (ER) stress is a critical positive factor deeply involved in cardiomyocyte apoptosis occurring in cardiovascular diseases including MI,^5^, ^6^, ^7^, ^8^ rendering it as a potential therapeutic target in MI.^9^ ER stress can induce cardiac dysfunction via architectural modifications and altering mitochondrial function in cardiomyocytes.^10^ Lately, the NADPH oxidase,^11^ calpain‐1^12^ and sestrin2‐mTORC1 signalling^13^ have been implicated in ER stress modulation in MI. However, how ER stress is regulated is not fully understood.

The fatty acid‐binding protein‐4 (FABP4), a member of the intracellular lipid‐binding protein family, is known to be responsible for transporting fatty acids.^14^ Studies have reported that FABP4 is connected with ER stress–related apoptosis in several circumstances. For instance, FABP4 mediates apoptosis via ER stress in mesangial cells of diabetic nephropathy.^15^ And exogenous FABP4 induces ER stress and apoptosis in HepG2 liver cells.^16^ Moreover, the silencing of FABP4 reduces hypoxia/reoxygenation injury through attenuating ER stress–mediated apoptosis.^17^ Yet, as far as we know, whether and how FABP4 modulates cardiomyocyte apoptosis are still not clear.

In this report, by investigating an in vitro hypoxia‐induced model, we show that FABP4 is induced in H9c2 cardiomyocytes following hypoxia exposure and that the FABP4 inhibitor BMS309403 can prevent hypoxia‐induced cardiomyocyte apoptosis, which is dependent on attenuating ER stress, thus recovering this novel role and mechanism of FABP4 in ER stress regulation in this condition.

## 2 MATERIALS AND METHODS

### 2.1 Cell culture and hypoxia induction

The embryonic rat heart‐derived H9c2 cardiomyocytes were obtained from the American Type Culture Collection (Manassas, VA, USA). H9c2 cardiomyocytes were maintained in DMEM supplemented with 10% foetal bovine serum at 37°C in a humidified atmosphere with 5% CO_2_ and 95% air. The hypoxia stimulation in vitro model was established by culturing cells in serum‐free DMEM medium placed in an anaerobic chamber containing 5% CO_2_, 94% N_2_ and 1% O_2_. H9c2 cardiomyocytes cultured in serum‐free DMEM placed in a normoxic incubator were used as normoxia controls. The study was approved by the Ethics Committee of The First Affiliated Hospital of Zhengzhou University.

### 2.2 Quantitative real‐time PCR analysis

The isolated total RNA from cultured H9c2 cardiomyocytes using the TRIzol reagent (Invitrogen, Carlsbad, CA, USA) was utilized to synthesize cDNA with the SuperScript III Reverse Transcriptase Kit (Invitrogen) according to the manufacturer's instructions. The quantitative real‐time PCR (qRT‐PCR) analysis was then implemented using the SYBR Green PrimeScript RT‐PCR Kit (TaKaRa, Otsu, Shiga, Japan) and the 7500 Real‐Time PCR System (Applied Biosystems, Foster City, CA, USA). The comparative Ct (threshold cycle) values were used to quantify mRNA levels. β‐actin is the internal control. Each PCR was performed with three replicates. The mRNA levels were calculated using the 2^−ΔΔCt^ method^18^ and presented as fold changes relative to the control. The PCR primer sequences for amplifying FABP4 transcripts and genomic fragments are listed as follows: *FABP4* forward 5′‐AGAAGTGGGAGTTGGCTTCG‐3′; *FABP4* reverse 5′‐ACTCTCTGACCGGATGACGA‐3′; *FABP4*‐HRE1 forward 5′‐GAGGCTCGCAATGGCTTAGG‐3′; *FABP4*‐HRE1 reverse 5′‐GCCCCTGTGTACTGCCAATG‐3′; *FABP4‐*HRE2 forward 5′‐GGTTTGTACTGCCCTGTCCT‐3′; *FABP4‐*HRE2 reverse 5′‐GTGGAGTCTGGAGCTCTTTCC‐3′; *FABP4‐*HRE3 forward 5′‐CTACAATGCTGTAGGAAAGAGC‐3′; *FABP4‐*HRE3 reverse 5′‐AACACACACACACACACAC‐3′.

### 2.3 Western blot analysis

The extracted total protein from cultured H9c2 cardiomyocytes using the RIPA lysis buffer (Beyotime, Shanghai, China) on ice was subjected to the sodium dodecyl sulphate‐polyacrylamide gel electrophoresis and then transferred onto a polyvinylidene difluoride membrane (Millipore, Billerica, MA, USA). The membrane was blocked with 5% non‐fat milk for 1 hour at room temperature, followed by incubation with primary antibodies (1:1000) at 4°C overnight. Afterwards, the membrane was incubated with horseradish peroxidase–conjugated secondary antibodies (1:5000) for another 1 hour at room temperature. The protein images were developed with the ECL Western Blotting Substrate (Pierce, Rockford, IL, USA). β‐actin is the loading control throughout. The protein expression was quantified with the ImageJ software (National Institutes of Health, USA).^19^ The sources of antibodies are listed as follows: anti‐FABP4 (Abcam, Cambridge, MA, USA, ab66682), anti‐β‐actin (Abcam, ab227387), anti‐HIF‐1α (Abcam, ab2185), Bax (Novus, Littleton, Colorado, USA, NBP1‐28566), Bcl‐2 (Santa Cruz, sc‐7382), cleaved caspase‐3 (Cell Signaling, Beverly, Massachusetts, USA, #9661), GPR78 (Novus, NLS3278), CHOP (Proteintech, Rosemont, IL, USA, 15204‐1‐AP), caspase‐12 (Cell Signaling, #2202), p‐PERK (Cell Signaling, #3179), PERK (Cell Signaling, #3192), p‐eIF2α (Cell Signaling, #9721), eIF2α (Cell Signaling, #9722) and ATF4 (Novus, NBP2‐42176).

### 2.4 Immunofluorescence

H9c2 cardiomyocytes seeded on coverslips were fixed using 4% paraformaldehyde for 10 minutes at room temperature and incubated with FABP4 antibodies (Invitrogen, PA5‐87384, 1:200) at 4°C overnight. At next day, cells were washed with phosphate‐buffered saline and incubated with the goat anti‐rabbit IgG (H + L) highly cross‐adsorbed secondary antibody Alexa Fluor Plus 594 (Invitrogen, A32740, 1:200) for 1 hour at 37°C. Nuclei were stained with 4',6‐diamidino‐2‐phenylindole (DAPI) for 5 minutes at room temperature. The fluorescence was captured using a Zeiss LSM 700 confocal fluorescence microscopy (Carl Zeiss, Oberkochen, Germany). The fluorescence intensity of five random fields of each group was analysed by the ImageJ software (National Institutes of Health, USA).^19^

### 2.5 Luciferase reporter assay

The fragments of FABP4 promoter containing the hypoxia‐responsive element were amplified by PCR and then cloned into the pGL3‐Basic vector (Promega, Madison, WI, USA) to generate the luciferase reporter constructs. These constructs were separately transfected into H9c2 cardiomyocytes using the Lipofectamine 3000 reagent (Invitrogen) according to the manufacturer's instruction. One day later, H9c2 cardiomyocytes were cultured under normoxia or hypoxia condition for another 24 hours. The luciferase activity was measured in each sample using the dual‐luciferase reporter assay system (Promega). The Renilla luciferase activity was used as an internal transfection control.

### 2.6 Chromatin immunoprecipitation assay

The chromatin immunoprecipitation (ChIP) assay was conducted as described previously.^20^ Briefly, H9c2 cardiomyocytes were cross‐linked for 10 minutes in 1% formaldehyde, and supernatants were collected by sonication in lysis buffer and mixed with the protein A Sepharose beads (Merck, Kenilworth, NJ, USA) on a rotator for 1 hour at 4°C. Then, amount of 5 µg anti‐HIF‐1a (Abcam) or rabbit isotype IgG antibody (Abcam) was added into the mixture and rotated for 16 hours at 4°C. The immunoprecipitates were eluted, and DNA was extracted by phenol/chloroform extraction and ethanol precipitation. The FABP4 promoter regions containing the hypoxia‐responsive element were quantified by qRT‐PCR analysis. The results are shown as relative to those of input samples.

### 2.7 Annexin V/PI double staining

The apoptosis of H9c2 cardiomyocytes was determined by annexin V‐FITC and PI double‐staining assay using the FITC Annexin V Apoptosis Detection Kit I (BD Pharmingen, San Jose, CA, USA) according to the manufacturer's protocols. The fluorescence was measured by a FACSCalibur flow cytometry (Becton‐Dickinson, Franklin Lakes, NJ, USA). The annexin V‐positive cells are defined as apoptotic cells.

### 2.8 Statistical analysis

All values are represented as means ± SD Statistical analysis was performed using the ANOVA test or Student's *t* test with the statistical package SPSS (version 18). Differences were considered significant when *P* < 0.05.

## 3 RESULTS

### 3.1 FABP4 is up‐regulated in hypoxia‐stimulated cardiomyocytes

**FIGURE 1:**
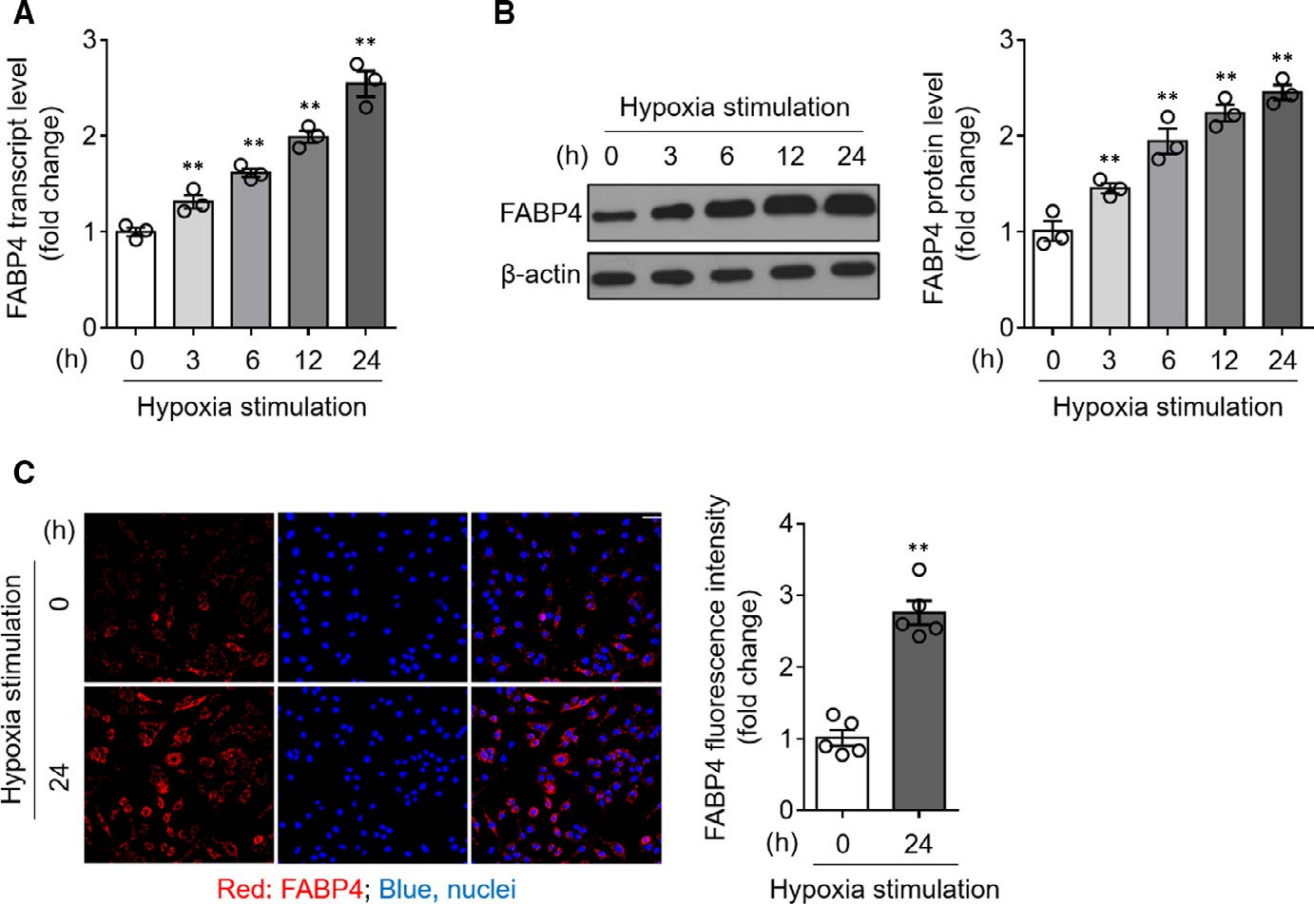
FABP4 expression is induced in cardiomyocytes exposed to hypoxia. (A, B) H9c2 cardiomyocytes were subjected to hypoxia as indicated for increasing periods of time. Normoxia (0 h) is the control treatment. The mRNA level (A) and protein expression (B) of FABP4 in harvested H9c2 cardiomyocytes were assessed by the quantitative real‐time PCR (qRT‐PCR) analysis and Western blot analysis, respectively. β‐actin is the internal control for FABP4 expression. One‐way analysis of variance (ANOVA) (n = 3). **, *P* < 0.01. (C) H9c2 cardiomyocytes were cultured under normoxia condition (0 h) or subjected to hypoxia for 24 h. FABP4 expression was visualized by the immunofluorescence method using the fluorescent‐labelled antibody. The nuclei were stained with DAPI. Scale bar, 100 µm. The intensity of FABP4 fluorescence was quantified by ImageJ. Student's *t* test (n = 5). **, *P* < 0.01

To understand whether FABP4 plays a role in cardiomyocyte apoptosis associated with ischaemic condition of MI, we applied an in vitro hypoxia‐induced injury model, in which H9c2 cardiomyocytes were cultured deprived of serum in an anaerobic chamber, containing 5% CO_2_, 94% N_2_ and 1% O_2_, to induce hypoxia‐induced apoptosis.^21^ Firstly, the quantitative real‐time PCR (qRT‐PCR) analysis showed that FABP4 transcript was prominently elevated time‐dependently in hypoxia‐exposed cardiomyocytes compared with those cultured under normoxia condition (Figure 1A). Consistent with this, FABP4 protein expression was also significantly up‐regulated in hypoxia‐exposed cardiomyocytes (Figure 1B). Moreover, similarly, the FABP4 up‐regulation was further supported by the evidence obtained by immunofluorescent assay, showing predominantly cytoplasmic positioning of FABP4 in H9c2 cardiomyocytes (Figure 1C). Taken as a whole, these observations indicate that FABP4 expression is induced in H9c2 cardiomyocytes upon hypoxia treatment.

### 3.2 FABP4 is transcriptionally activated by HIF‐1α

**FIGURE 2:**
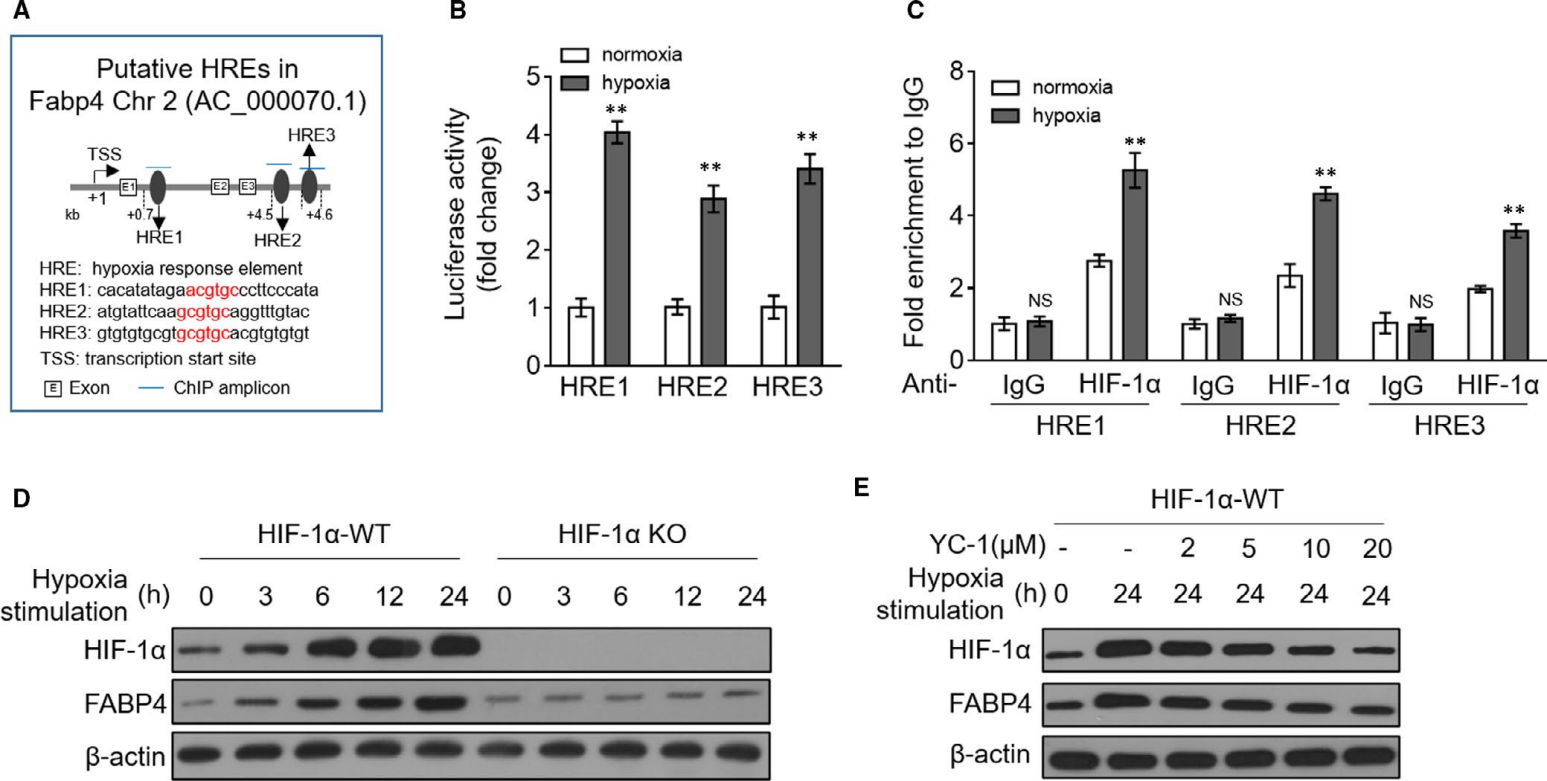
HIF‐1α transcriptionally activates FABP4. (A) The putative HIF‐1a‐responsive elements (HREs) in FABP4 gene. (B) The relative luciferase activity of FABP4 gene luciferase constructs transfected in H9c2 cardiomyocytes cultured under normoxia or hypoxia condition for 24 h. Student's *t* test (n = 5). **, *P* < 0.01. (C) H9c2 cardiomyocytes were cultured under normoxia or hypoxia condition for 24 h. The chromatin immunoprecipitation (ChIP) assay using the HIF‐1a antibody was conducted to determine HIF‐1a binding to the FABP4 region containing putative HREs. The precipitated DNA samples were analysed by qRT‐PCR assay. Student's *t* test (n = 3). **, *P* < 0.01. NS, not significant. (D) HIF‐1a wild‐type (WT) or knockout (KO) H9c2 cardiomyocytes were subjected to hypoxia as indicated for increasing periods of time. Normoxia is the control treatment. The protein expression of FABP4 and HIF‐1a was measured by Western blot analysis. β‐actin is the internal control. (E) HIF‐1a‐WT H9c2 cardiomyocytes were cultured under normoxia or hypoxia condition for 24 h in the presence or absence of treatment of increasing concentrations of YC‐1. FABP4 and HIF‐1a expressions were determined by Western blot analysis. Images were representative of three independent experiments

It has been reported that FABP4 is a hypoxia‐inducible gene in hepatic cells^22^ and placenta.^23^ This is in consistence with our findings observed in H9c2 cardiomyocytes (Figure 1). However, the mechanism underlying FABP4 induction in this scenario is not clear. Through using the MatInspector program,^24^ we inspected FABP4 gene sequence and three putative hypoxia response elements (HREs) were identified (Figure 2A), as predicted by the consensus binding sequence, (AG)CGT(GC)C.^25^ Next, the luciferase reporter assay showed that the luciferase activity of constructs containing three individual HREs was highly induced in hypoxia‐exposed H9c2 cardiomyocytes (Figure 2B). In addition, HIF‐1a was recruited to three FABP4 gene regions encompassing corresponding HREs, which was further enhanced upon hypoxia, as demonstrated by the ChIP experiment (Figure 2C). Further, in HIF‐1a knockout H9c2 cardiomyocytes, FABP4 failed to be up‐regulated upon hypoxia (Figure 2D). In agreement with these data, hypoxia‐induced FABP4 induction was overtly suppressed when H9c2 cardiomyocytes were treated with HIF‐1a inhibitor YC‐1 (Figure 2E). Therefore, these data illustrate that FABP4 is a hypoxia‐inducible gene as a direct target of HIF‐1a, which activates its transcription in H9c2 cardiomyocytes under hypoxia condition.

### 3.3 Pharmacological inhibition of FABP4 protects against hypoxia‐induced cardiomyocyte apoptosis

**FIGURE 3:**
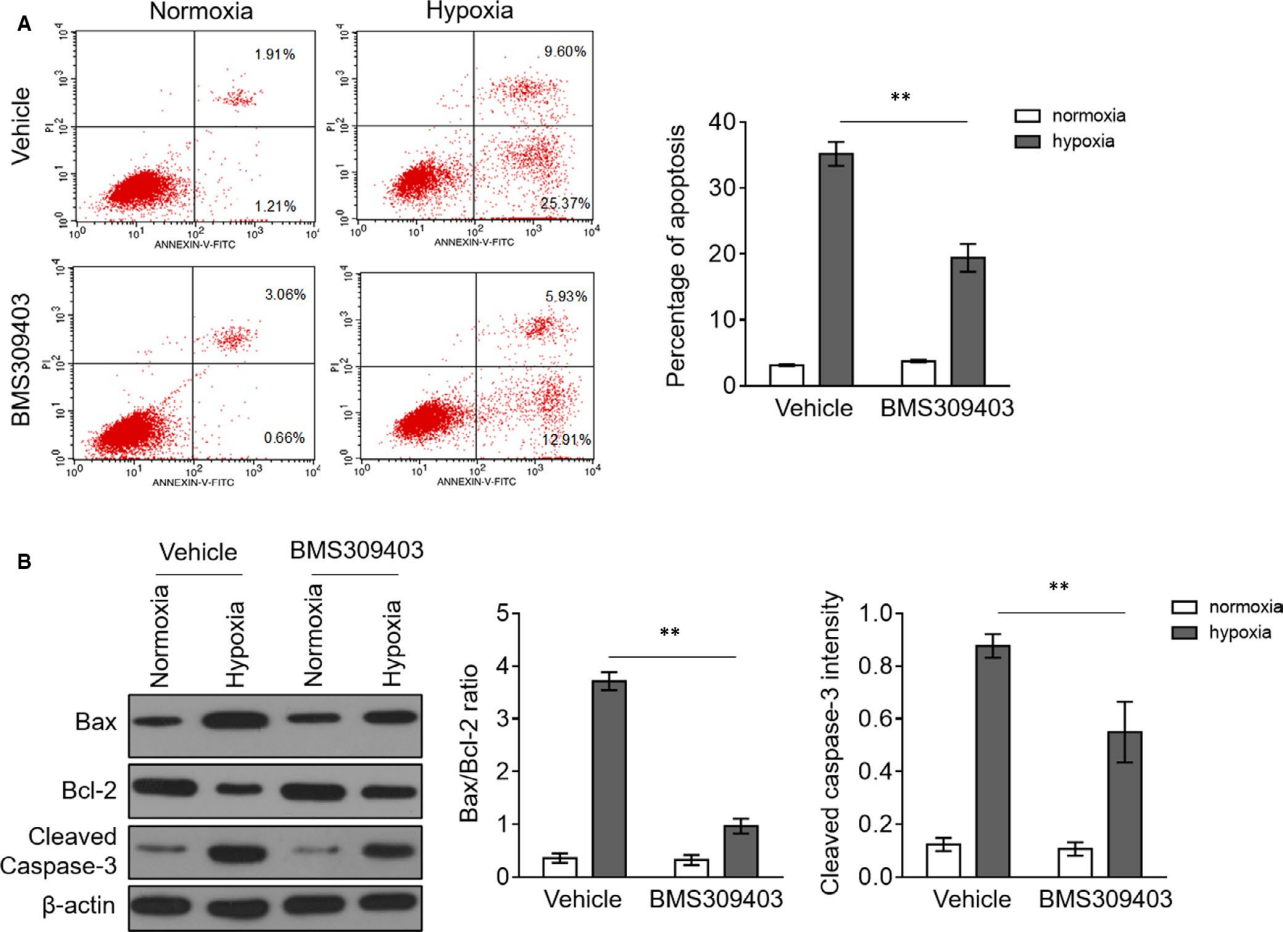
FABP4 inhibitor BMS309403 protects against hypoxia‐induced apoptosis in cardiomyocytes. (A) H9c2 cardiomyocytes were cultured under normoxia or hypoxia condition for 24 h in the presence or absence of 50 µM BMS309403. The cell apoptosis was evaluated by the flow cytometric analysis combined with annexin V‐FITC and PI double‐staining assay. The annexin V‐positive cells are defined as apoptotic cells, and the percentage of apoptotic cells is depicted in the right. Student's *t* test (n = 4). **, *P* < 0.01. (B) H9c2 cardiomyocytes were treated as in (A). The protein expressions of Bax, Bcl‐2 and cleaved caspase‐3 were determined by Western blot analysis. β‐actin is the internal control. The quantification analysis of protein expression is shown in the right. Student's t test (n = 3). **, *P* < 0.01

FABP4 up‐regulation in hypoxia‐exposed H9c2 cardiomyocytes motivated us to examine whether it affects hypoxia‐induced apoptosis, to some extent mimicking ischaemia‐induced cardiomyocyte apoptosis in MI.^21^, ^26^ To investigate this issue, we applied a strategy to inhibit FABP4 activity via administrating BMS309403, a potent and selective small‐molecule inhibitor of FABP4.^27^ As shown by annexin V‐FITC and PI double‐staining assay, BMS309403 did not significantly affect H9c2 cardiomyocyte apoptosis under normoxia condition, but it largely restricted apoptosis of hypoxia‐exposed H9c2 cardiomyocytes (Figure 3A), proving that FABP4 inhibitor BMS309403 can prevent apoptosis induced by hypoxia. To verify this concept, we then checked the expressions of typical apoptotic markers, including Bax, Bcl‐2 and cleaved caspase‐3. The result turned out to be that hypoxia treatment indeed caused remarkable increase in Bax/Bcl‐2 ratio and cleaved caspase‐3 expression, indicating the induction of apoptosis in H9c2 cardiomyocytes, and adversely, this phenotype was prominently attenuated in the presence of BMS309403 (Figure 3B), demonstrating that FABP4 activity inhibition via BMS309403 minimizes cardiomyocyte apoptosis aroused by hypoxia. In other words, these results suggest that FABP4 induction plays a negative role in cardiomyocyte survival in response to hypoxic insults.

### 3.4 FABP4 inhibitor attenuates ER stress in hypoxia‐exposed cardiomyocytes

**FIGURE 4:**
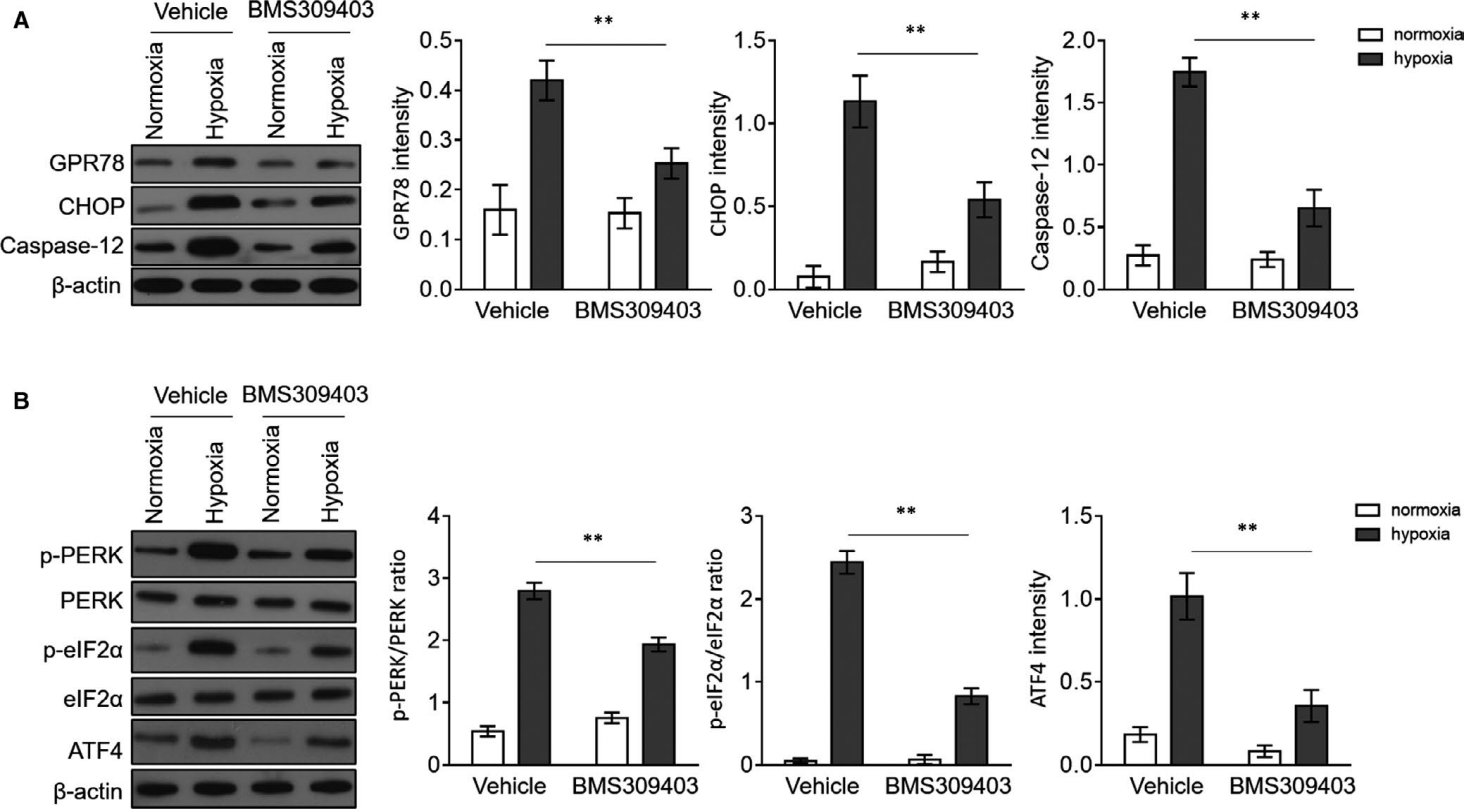
FABP4 inhibitor BMS309403 attenuates ER stress in cardiomyocytes exposed to hypoxia. (A, B) H9c2 cardiomyocytes were cultured under normoxia or hypoxia condition for 24 h in the presence or absence of 50 µM BMS309403. The protein expressions of GPR78, CHOP and caspase‐12 (A), and p‐PERK, PERK, p‐eIF2α, eIF2 and ATF4 (B) were determined by Western blot analysis. β‐actin is the internal control. The quantification analysis of protein expression is shown in the right. Student's *t* test (n = 3). **, *P* < 0.01

To gain a mechanistic insight into BMS309403 protection against hypoxia‐induced cardiomyocyte apoptosis, we subsequently investigated the involvement of ER stress alteration in influencing this biological process, as FABP4 has been implicated in modulating ER stress,^16^, ^28^ a pivotal contributing factor to apoptosis.^29^ To this end, we monitored the expression changes of ER stress markers by Western blot analysis, such as the G protein–coupled receptor 78 (GPR78), CCAAT‐enhancer‐binding protein homologous protein (CHOP) and caspase‐12. As a result, we found that hypoxia stimulation augmented the expressions of GPR78, CHOP and caspase‐12 in H9c2 cardiomyocytes, but BMS309403 treatment evidently lowered their expressions (Figure 4A), illustrating that FABP4 inhibition attenuates hypoxia‐promoted ER stress and apoptosis. These data again are in line with the results presented in Figure 3, because caspase‐12 is essential for initiating apoptosis induced by ER stress.^30^ Furthermore, we also measured the expressions of key molecules imbedded in the protein kinase R‐like endoplasmic reticulum kinase (PERK)/eukaryotic initiation factor‐2 alpha (eIF2α)/activating transcription factor‐4 (ATF4) pathway, which is important for cellular adaptation to apoptosis elicited from hypoxia‐induced ER stress.^31^ Consistent with the alteration of GPR78/caspase‐12 pathway (Figure 4A), the hypoxia‐activated PERK/eIF2α/ATF4 signalling pathway similarly displayed pronounced reduction by BMS309403 treatment, as indicated by the decreases in p‐PERK/PERK, p‐eIF2α/eIF2α and ATF4 expression (Figure 4B). Hence, FABP4 inhibitor BMS309403 counteracts hypoxia‐ER stress in H9c2 cardiomyocytes.

### 3.5 Activating ER stress abrogates protective effects of BMS309403 on hypoxia‐induced apoptosis in cardiomyocytes

**FIGURE 5:**
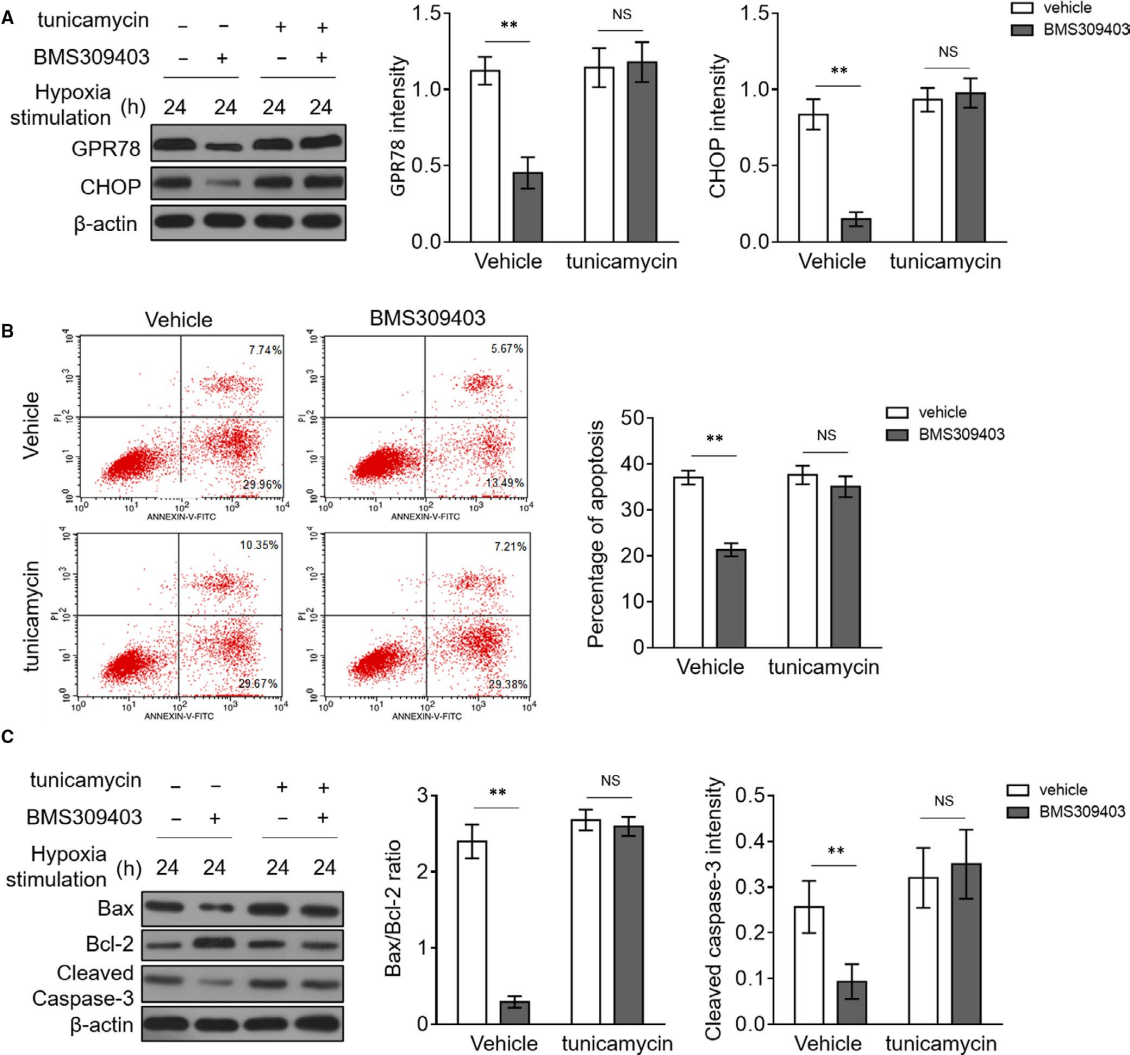
ER stress activation abrogates protection of FABP4 inhibition against hypoxia‐induced apoptosis in cardiomyocytes. (A‐C) H9c2 cardiomyocytes were cultured under hypoxia condition in the presence or absence of 50 µM BMS309403 and 10 µM tunicamycin as indicated for 24 h. (A) The protein expressions of GPR78 and CHOP were determined by Western blot analysis. β‐actin is the internal control. The quantification analysis of protein expression is shown in the right. Student's t test (n = 3). **, *P* < 0.01. NS, not significant. (B) The cell apoptosis was assessed by the flow cytometric analysis combined with annexin V‐FITC and PI double‐staining assay. The annexin V‐positive cells are defined as apoptotic cells, and the percentage of apoptotic cells is depicted in the right. Student's *t* test (n = 4). **, *P* < 0.01. NS, not significant. (C) The protein expressions of Bax, Bcl‐2 and cleaved caspase‐3 were determined by Western blot analysis. β‐actin is the internal control. The quantification analysis of protein expression is shown in the right. Student's *t* test (n = 3). **, *P* < 0.01. NS, not significant

Finally, to prove whether there exists a potential causal link between BMS309403 restriction on ER stress accumulation and its protection against hypoxia‐induced apoptosis in cardiomyocytes, we took advantage of tunicamycin, a commonly used activator of ER stress,^32^ aimed at reversing BMS309403‐restricted ER stress in H9c2 cardiomyocytes under hypoxic stimulation. As expected, tunicamycin combination treatment indeed rescued ER stress, which was invariably lowered by BMS309403, as demonstrated by the expression changes of GPR78 and CHOP (Figure 5A). More significantly, keeping along with the recovered ER stress, tunicamycin treatment resulted in drastically reversed hypoxia‐induced cardiomyocyte apoptosis, which was prevented by MS309403 (Figure 5B). This demonstrates that BMS309403 limits hypoxia‐induced apoptosis through lowering ER stress. Moreover, in order to reinforce this conclusion, we interrogated the expression alterations of Bax, Bcl‐2 and cleaved caspase‐3. In concert, Western blot analysis revealed that BMS309403‐decreased Bax/Bcl‐2 ratio and cleaved caspase‐3 expression were all recovered to the extent of control group in the presence of tunicamycin (Figure 5C), further strengthening the concept that the negative regulation of ER stress by FABP4 inhibitor BMS309403 underlies its protective function against hypoxia‐induced cardiomyocyte apoptosis. Overall, these data strongly suggest that FABP4, a fatty acid transporter induced in a HIF‐1a‐dependent manner by hypoxia, acts to elevate ER stress to lead to cardiomyocyte apoptosis.

## 4 DISCUSSION

**FIGURE 6:**
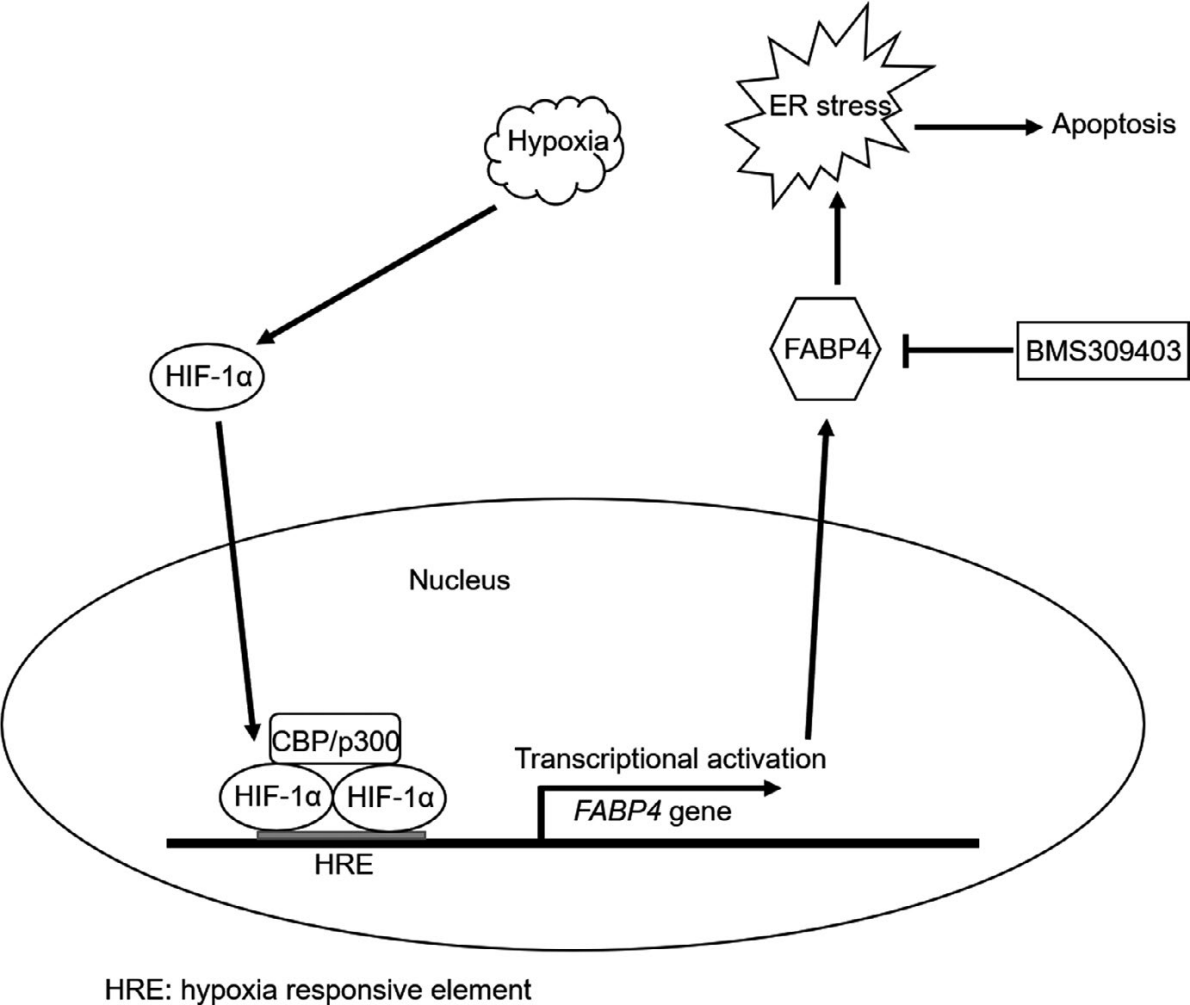
A schematic model of this study. FABP4 is transcriptionally induced by HIF‐1α upon hypoxia stimulation. FABP4 is about to elevate ER stress leading to apoptosis, which is blocked by inhibitor BMS309403

In the current study, we explored the role and molecular mechanism of FABP4 involved in hypoxia‐induced cardiomyocyte apoptosis using an in vitro experimental model. Meanwhile, we also discovered the HIF‐1a‐regulated FABP4 transcriptional activation in this process. By exploiting a strategy of FABP4 activity inhibition with its pharmacological inhibitor BMS309403, we related FABP4 function to the regulation of cardiomyocyte apoptosis and ER stress, and further established that these two activities of FABP4 are mechanistically connected tightly, in which the FABP4‐dysregulated ER stress contributes to cardiomyocyte apoptosis under hypoxia stimulation. Thus, our research may uncover FABP4 as a novel regulator of cardiomyocyte apoptosis associated with MI pathogenesis, and provide a molecular basis for applying FABP4 activity inhibitor in the intervention of cardiomyocyte loss during MI, such as BMS309403 (Figure 6).

FABP4 is also known as aP2, a cytoplasmic fatty acid chaperone, previously found to be primarily expressed in adipocytes and myeloid cells.^33^ As a supplement, we report here that FABP4 is expressed in H9c2 cardiomyocytes and interestingly that its expression can be induced under hypoxic condition. By analysing the FABP4 gene and performing luciferase reporter assay, we identified FABP4 as a hypoxia‐inducible gene in H9c2 cardiomyocytes. In truth, analogical with our results, FABP4 was similarly shown to be a hypoxia‐inducible gene in the liver, which sensitizes mice to liver ischaemia/reperfusion injury.^22^ Additionally, in the placenta, the HIF‐1a and HIF‐2a possess the ability to regulate FABP4 expression.^23^ Relevantly, in our ChIP assay and experiments complying with the loss‐of‐function strategy, we show that HIF‐1a is required for FABP4 transcriptional activation in hypoxia‐exposed H9c2 cardiomyocytes. It seems that this FABP4 induction is solely relied on HIF‐1a, as, in HIF‐1a knockout cells, this phenomenon completely vanished. Nevertheless, whether HIF‐2a and other transcriptional factors, such as FOXO1^34^ and PTEN,^35^ also participate in FABP4 regulation cannot be ruled out easily, because FOXO1 and PTEN are functionally connected to HIF‐1a and hypoxia.^36^, ^37^ Moreover, it should be noted that our discoveries were obtained from H9c2 cardiomyocytes in vitro only; whether and how FABP4 is modulated in other hypoxic experimental systems including animal models are largely uncertain. Interestingly, the circulating FABP4 is considered as a prognostic biomarker in patients with acute ischaemic stroke.^38^ According to our findings, it would be significant to check the expression patterns of FABP4 in tissue samples from MI patients compared with normal controls. These future research directions could offer more clinically relevant implications between FABP4 and MI pathogenesis.

FABP4 plays multiple and important roles in metabolic and cardiovascular diseases, such as diabetes mellitus, cardiac dysfunction and atherosclerosis.^39^ Lately, new roles in cancers ^40^ and osteoarthritis ^41^ have also been associated with FABP4, indicating the versatile activities of FABP4. In recent years, FABP4 was found to mediate apoptosis in mesangial cells via controlling ER stress,^15^ and exogenous FABP4 was found to induce ER stress in HepG2 liver cells.^16^ Conversely, FABP4 inhibition with BMS309403 reduces ER stress and apoptosis in hypoxia followed by reoxygenation (HR)‐induced HK‐2 cells.^42^ Consistent with these literatures, we observed that the pharmacological inhibition of FABP4 using BMS309403 protected against apoptosis and attenuated ER stress in hypoxia‐exposed cardiomyocytes. As known, if ER stress is prolonged due to failed adaptive unfolded protein response (UPR), mediated by pathways including the PERK/eIF2α/ATF4 signalling, apoptosis will ensue following the activation of GRP78/caspase‐12.^43^ Keeping pace with this knowledge, we provided data showing that tunicamycin elevated ER stress and simultaneously rescued BMS309403 effects on hypoxia‐induced cardiomyocyte apoptosis, therefore proving that BMS309403 attenuates hypoxia‐induced cardiomyocyte apoptosis through reducing ER stress. In a previous report, FABP4 silencing attenuated ER stress–mediated apoptosis by activating PI3K/Akt pathway.^17^ Besides, inactivated PI3K/Akt results in CHOP induction that causes ER stress–mediated cell death.^44^ Based on these clues, we reason that the PI3K/Akt pathway is presumably the intermediary mechanism underlying BMS309403‐lowered ER stress. Further investigations would be required to address whether this is the case. The dysregulated ER stress is deeply connected with hypoxia‐induced injury, including the initiation of apoptosis, which can be promoted in this scenario by several means, such as the transcriptional activation of CHOP/GADD153, the caspase 12‐dependent or the c‐Jun NH2‐terminal kinase (JNK)‐dependent pathway.^45^ The activation of CHOP and caspase‐12 is undoubtedly involved in BMS309403‐modulated hypoxia‐induced injury according to our available data. But, whether and how the JNK‐dependent pathway contributes to BMS309403 activity need further investigations.

In conclusion, in the light of the evidence we present at the current stage, we suppose that down‐regulating ER stress via inhibiting FABP4 activity, at least using BMS309403, might have alleviating effects to reduce ischaemia‐induced cardiomyocyte loss in the pathogenic process of MI.

## CONFLICT OF INTEREST

None declared.

## AUTHOR CONTRIBUTIONS

**Fuqiang Sun:** Conceptualization (lead); Data curation (lead); Writing‐original draft (lead); Writing‐review & editing (lead). **Jiangchuan Du:** Methodology (lead). **Hongbin Li:** Software (lead). **Shuang Hao:** Visualization (equal). **Guochang Zhao:** Investigation (equal). **Fanfan Lu:** Supervision (equal).

## Data Availability

The data would be available from the correspondence author on reasonable request.
